# COMORBIDITY IN NEURODEGENERATIVE DISEASES AND MENTAL HEALTH CONDITIONS: IMPLICATIONS FOR HEALTH SYSTEM BURDEN

**DOI:** 10.1093/geroni/igz038.057

**Published:** 2019-11-08

**Authors:** Colleen J Maxwell, Laura C Maclagan, Ruth Ann Marrie, Luke Mondor, Walter Wodchis, David B Hogan, Susan E Bronskill

**Affiliations:** 1 University of Waterloo, Waterloo, Ontario, Canada; 2 ICES, Toronto, Ontario, Canada; 3 University of Manitoba, Winnipeg, Manitoba, Canada; 4 Institute of Health Policy, Management & Evaluation, University Of Toronto, Toronto, Ontario, Canada; 5 University of Calgary, Calgary, Alberta, Canada

## Abstract

Research suggests that older adults with neurodegenerative diseases are at increased risk of developing a subsequent neurodegenerative or comorbid psychiatric disorder or both. Depression and other psychiatric conditions, though prevalent, are often under-diagnosed and under-treated among those with neurodegenerative conditions potentially leading to more rapid disease progression, poorer health outcomes and increased health care use. Few population-based studies have comprehensively examined the risk and temporal ordering of common neurodegenerative and psychiatric conditions, including whether these associations differ by age or sex. Initial findings regarding the incidence of ordered pairs of neurological conditions (including dementia, Parkinson’s disease and stroke) and psychiatric disorders (including mood and anxiety disorders, and schizophrenia) will be summarized. This population-based retrospective cohort study will provide essential data to allow policymakers, planners and providers to better anticipate the prognosis and care needs of older adults with comorbid neurodegenerative and psychiatric conditions.

